# Extremely Low-Frequency Magnetic Fields and Redox-Responsive Pathways Linked to Cancer Drug Resistance: Insights from Co-Exposure-Based *In Vitro* Studies

**DOI:** 10.3389/fpubh.2018.00033

**Published:** 2018-02-23

**Authors:** Stefano Falone, Silvano Santini, Valeria Cordone, Giovanna Di Emidio, Carla Tatone, Marisa Cacchio, Fernanda Amicarelli

**Affiliations:** ^1^Department of Life, Health and Environmental Sciences, University of L’Aquila, L’Aquila, Italy; ^2^Department of Neurosciences, Imaging and Clinical Sciences, University “G. d’Annunzio”, Chieti, Italy; ^3^Institute of Translational Pharmacology (IFT)—National Research Council (CNR), L’Aquila, Italy

**Keywords:** chemoresistance, reactive oxygen species, glutathione, cytotoxicity, genotoxicity, catalase, peroxidase, cancer stem cell

## Abstract

Electrical devices currently used in clinical practice and common household equipments generate extremely low-frequency magnetic fields (ELF-MF) that were classified by the International Agency for Research on Cancer as “possible carcinogenic.” Assuming that ELF-MF plays a role in the carcinogenic process without inducing direct genomic alterations, ELF-MF may be involved in the promotion or progression of cancers. In particular, ELF-MF-induced responses are suspected to activate redox-responsive intracellular signaling or detoxification scavenging systems. In fact, improved protection against oxidative stress and redox-active xenobiotics is thought to provide critical proliferative and survival advantage in tumors. On this basis, an ever-growing research activity worldwide is attempting to establish whether tumor cells may develop multidrug resistance through the activation of essential cytoprotective networks in the presence of ELF fields, and how this might trigger relevant changes in tumor phenotype. This review builds a framework around how the activity of redox-responsive mediators may be controlled by co-exposure to ELF-MF and reactive oxygen species-generating agents in tumor and cancer cells, in order to clarify whether and how such potential molecular targets could help to minimize or neutralize the functional interaction between ELF-MF and malignancies.

## Introduction

Cancer is characterized by an unrestrained proliferation of cells that are functionally and morphologically different (1). Chemotherapy often fail to completely eradicate cancers due to the development of multidrug resistance (MDR), and this represents a major obstacle to the effective treatment of human malignancies, which currently cause approximately 7.5 million deaths per year worldwide (2). Cell cultures allowed an extensive study of the phenomenon *in vitro*, and cell lines have been widely used to explore the mechanisms involved in MDR, however, the mechanisms by which tumors become chemoresistant still remain largely unclear (3). Cancer cell fate is strictly dependent on reactive oxygen species (ROS) metabolism, as both cell proliferation and differentiation are under the control of redox homeostatic balance (4). However, ROS can act as a double-edged sword, and ROS in excess are a known cause of oxidative damage to vital cellular macromolecules, such as DNA, proteins, and lipids (5). In tumor cells, ROS production and oxidative stress usually occur following exposure to chemotherapeutic drugs (e.g., doxorubicin, vinblastine, and platinum compounds) (6, 7). Therefore, not unexpectedly, the enhancement of major detoxification and antioxidant activities is among the major mechanisms on which anticancer resistance relies (8). Other means through which MDR may develop can involve strengthened proliferative activity, reduced responsiveness to cell death-promoting treatments, increased capacity of DNA damage repair, and improved drug efflux (8).

Many signaling pathways underlying changes in cancer cell behavior are tightly regulated by the redox milieu or by redox-responsive elements (9), and this grabbed the attentions of many biophysics research groups worldwide, in that several research team started to consider common physical pollutants like electromagnetic fields as potential hazardous agents due to their known ability to induce redox alteration within cells and tissues (10–13). Unlike electric fields (EF), magnetic fields (MF) penetrate into human tissues without any substantial intensity decrement (14). For this reason, extremely low-frequency magnetic fields (ELF-MF), with common frequencies of 50–60 Hz and flux densities up to 100 Gauss, that are typically originated by distribution of electricity, industrial equipments, domestic appliances, and medical instruments, came under the magnifying glass of the biophysics community (7). Indoor domestic environments are characterized by ELF-MF flux densities of few microtesla (15–17), even though some occupational roles require significant amounts of time spent near transformers and electricity distribution lines, which are characterized by much higher ELF-MF intensities (18). Sixteen years ago, the World Health Organization’s International Agency for Research on Cancer officially established that ELF-MF may represent a possible carcinogenic hazard for humans (19). Specifically, ELF-MF are suspected to play a significant role in co-carcinogenesis, as well as in the progression of tumorigenesis (20–24), during which malignant cells are believed to acquire crucial survival capacity or proliferative advantage (25–27). In particular, later stages of malignancies are often linked to both metabolic reprogramming, enhanced detoxification capacity, and high-DNA damage repairing activities, which, in turn, may result in drug resistance and poor clinical outcome (9, 25–27).

Extremely low-frequency fields have been described as agents with a specific capacity to promote free radical production and alterations in the redox homeostasis (10). This arose the question as to whether and how ELF fields could trigger relevant changes in malignant cell behavior. In particular, an emerging interest of biomedical researchers worldwide is focusing on the possible effects of ELF-MF on biomolecular mechanisms underlying resistance toward anticancer treatments. Such attention was strengthened by some interesting pioneering *in vivo* studies in which chemotherapeutic agents and electromagnetic fields were utilized to detect possible synergistic or antagonistic interactions (28, 29).

Since ELF field-generating medical equipments are increasingly used for the clinical treatment of oncology patients in intensive care units (30), over the past 25 years researchers have been engaged in conducting extensive investigations on how ELF-MF may impact cell behavior and biomolecular phenotype (31–36). In this regard, some researchers presented interesting findings with regard to ELF-MF exposure-derived effects on cancer cell biology or metabolism (35, 37–39). Interestingly, the exposure to 50/60 Hz MF has been found to promote changes in signal transduction pathways that are known to be directly involved in proliferative processes (40–44). Those signaling pathways, such as the mitogen-activated protein kinases—extracellular-signal-regulated kinase 1/2 and p38, also respond to physical stressors such as UV radiation and thermal shock (45).

However, many of the effects often appeared to be either quantitatively weak or questionable regarding their real biological relevance. Unfortunately, much less research activity was carried out by using co-exposure designs, with particular focus on *in vitro* paradigms that allow an accurate control of the experimental conditions and a reliable identification of the cellular and molecular working mechanisms underlying the ELF field-induced effects on either cancer cell behavior or resistance against relevant anticancer treatments.

This review attempts to give a brief summary of the PubMed- and Scopus-indexed reports available so far with regard to *in vitro* co-exposure-based interventional researches that were aimed at investigating the possible ELF-MF-induced changes in resistance or vulnerability of malignant cells toward well-known redox-active physical/chemical treatments.

Original articles were included in this review only if: (a) experiments were conducted using tumor/cancer cells and (b) simultaneous or sequential combined exposures to ELF-MF and chemical/physical agents were carried out. Exclusion criteria included: (a) cells were treated with ELF-MF only and (b) cells did not derive from tumors/cancer.

## ELF-MF-Related Effects on Cancer Cell Response to Differentiating Treatments

As reviewed by Peiris-Pagès et al. (1), the loss of specific tissue traits, along with de-differentiation and regression into a more primitive phenotype, is a peculiar feature of tumors. Indeed, pro-differentiating approaches are currently used as one of the most useful strategies to retard cancer in animals and humans (46–50).

Chen et al. (51) found that a 60 Hz, 4 µT ELF-MF inhibited differentiation of dimethyl sulfoxide- and hexamethylene bis-acetamide-treated erythroleukemia cells, and this was associated with the preservation of telomerase activity, whose expression sustains an undifferentiated cell status (52). High-risk neuroblastomas are often treated with retinoic acid (RA) or RA-derived compounds to induce growth arrest and cell differentiation (46, 47, 49, 53). In 2003, Pirozzoli and co-workers reported that the all-trans-retinoic acid (ATRA)-induced decrease in proliferation of human neuroblastoma LAN-5 cells was inhibited by a 72-h exposure to an MF of 50 Hz and 1 mT. Conversely, Marcantonio et al. (54) found that a mid-term exposure to a 50 Hz, 1 mT MF significantly potentiated the effects of ATRA treatment on human neuroblastoma cell line BE(2)C, by decreasing the proliferation rate and by inducing neurite outgrowth. Trillo et al. (55) gave evidence that the activation of proliferative response elicited by all-trans-retinol (ROL) in NB69 human neuroblastoma cells is potentiated by a 42-h exposure to a 50 Hz, 100 µT MF, whereas in HepG2 human hepatocarcinoma cells the ROL-induced hyperproliferative effect was significantly inhibited by the ELF-MF. The same authors showed that the combined treatment of NB69 neuroblastoma cells with ROL and 10–100 µT MF did not revert the ROL-dependent cell growth arrest (56). It should be noted that in their two studies the authors used very different ROL concentration, obtaining either hyperproliferative or hypoproliferative responses in NB69 cells with 0.5 and 2.0 µM ROL, respectively. Lastly, a 5-day 50 Hz, 1 mT ELF-MF was shown to enhance nerve growth factor-induced differentiation of rat pheochromocytoma tumor cells (57).

## ELF-MF-Related Effects on Cancer Cell Response to Cytostatic/Cytotoxic Treatments

Resistance to cell death is an important aspect of tumorigenesis (58). Therefore, a possible alteration of cellular responses to pro-differentiating or cytotoxic/cytostatic treatments could be a possible means through which cancer cell phenotype may be altered by ELF-MF.

In 2002, Ruiz-Gómez et al. demonstrated that a 1-h exposure to a pulsed 1–25 Hz, 1.5 mT ELF field potentiated the cytotoxic effect of mitomycin C, vincristine, and cisplatin, three antiblastic chemicals that are frequently used in cancer chemotherapy, in multiresistant human colon adenocarcinoma cells. Ding et al. (59) found that 24 h in the presence of 60 Hz, 5 mT potentiated stimulation of H_2_O_2_-dependent apoptosis in human leukemia HL-60 cells. Jian et al. (60) showed that an intermittent treatment with 100 Hz, 0.7 mT MF increased the apoptotic rate of human hepatoma cells exposed to low-dose X-ray radiation. In addition, Wócik-Piotrowicz et al. (61) showed in human cells isolated from histiocytic lymphoma that a short-term exposure to 50 Hz, 6.5 mT with static MF amplified the loss of cell viability caused by puromycin (PMC), a known unselective cell death inducer (62). Kaszuba-Zwoinska et al. (63) reported that an acute exposure to a 50 Hz, 45 ± 5 mT pulsed MF significantly enhanced the apoptotic response of human acute monocytic leukemia MonoMac6 cells to PMC, colchicine, and cyclophosphamide. Baharara et al. (64) demonstrated that a 2-h 50 Hz, 20 µT increased sensitivity to apoptosis induced by cisplatin, a DNA-targeting and ROS-promoting agent used in chemotherapy (65), in human ovarian adenocarcinoma cells, and this was accompanied by a significant increase of activities of both caspase 3 and 9. Accordingly, 24 h of exposure to a 50 Hz, 1 mT ELF-MF enhanced caspase-dependent apoptosis caused by ROS-promoting MPP + (1-methyl-4-phenylpyridinium) in SH-SY5Y human neuroblastoma cells (66).

On the other hand, many researchers reported substantially different findings. Liburdy et al. (67) reported that a 60 Hz, 1.2 µT ELF-MF totally blocked the oncostatic action of melatonin on mammary adenocarcinoma cells. Harland and Liburdy (68) showed that a 1.2 µT, 60 Hz MF partially blocked inhibitory action of tamoxifen on the growth of mammary tumor MCF-7 cells for exposure durations greater than 6 days. Pirozzoli et al. (69) demonstrated that neuroblastoma LAN-5 cells are protected from campthotecin-induced apoptosis if the cytotoxic compound is used in combination with a 24-h treatment with 50 Hz, 1 mT ELF field. Girgert et al. (70) found that 7 days of 1.2 µT of a sinusoidal 50 Hz alternating MF potentiated the tamoxifen-induced cytoproliferative effect in a breast cancer cell line. In 2006, De Nicola and colleagues described how PMC-induced apoptosis in human lymphoblasts from histiocytic lymphoma was strongly limited by a 2-h treatment with a 0.1 mT ELF field, with such effect requiring reduced glutathione (GSH) as an essential means. Similarly, Palumbo et al. (71) demonstrated that the increase of caspase-3-dependent apoptosis induced by anti-Fas treatment was reduced of 22% when immortalized Jurkat leukemic cells were pre-exposed to a 1-h intermittent 50 Hz, 1 mT MF. Furthermore, Kaszuba-Zwoinska et al. (62) found that PMC-induced cell death of human lymphoblasts from histiocytic lymphoma was significantly inhibited by a pulsating ELF field of 50 Hz, 45 mT. Cid et al. (72) reported that the melatonin-induced antiproliferative response in HepG2 human hepatocarcinoma cells was totally abolished by a 42-h intermittent exposure to a 50 Hz, 10 µT MF. Moreover, an acute (10 min) exposure to a 217 Hz, 120 µT ELF-MF was found to reduce bleomycin-induced apoptotic response in human erythroleukemia K562 cells (73). Brisdelli et al. (74) observed increased expression of Bcl-2, a marker of anti-apoptotic response (75), in human K562 chronic myeloid leukemia cells simultaneously treated with quercetin and ELF-MF for 24 h, when compared with quercetin-treated and control cells. In the same study, 1–3 days of ELF-MF exposure were found to reduce the growth inhibition induced by quercetin treatment in leukemia cells, and this was associated to a concomitant reduction of levels of caspase-3 activity, along with decreased expression of anti-apoptotic proteins, such as Bcl-xL and Mcl-1 (74). Some of us also found that a chronic exposure to a 50 Hz, 1 mT MF protects neuroblastoma cells from cytotoxicity associated with methylglyoxal (MG), one of the major endogenous precursor of glycative and oxidative stress (76). In the same cell line, some of us have recently reported that long-term exposures to ELF-MF as low as 100 µT are able to abolish or limit the cytotoxic effect of both hydrogen peroxide and doxorubicin (77).

It should be noted that several papers reported no significant effects of ELF-MF on cytotoxic/cytostatic action of chemicals on malignant cells. In 2003, Laqué-Rupérez et al. demonstrated that a 25 Hz, 1.5 mT pulsed MF exposure did not affect the levels of methotrexate cytotoxicity in MCF-7 cancer cells. Others showed that neuroblastoma LAN-5 cells are protected from campthotecin-induced apoptosis if the cytotoxic compound is used in combination with a 24-h treatment with 50 Hz, 1 mT ELF field (69). Mizuno et al. (78) found no significant differences in survival rate when human lung-derived SV40-transformed cells were exposed to UV irradiation alone, when compared with cells exposed to both 24-h 60 Hz, 5 mT MF and UV radiation. In addition, Höytö et al. (79) recently showed that a 24-h exposure to an ELF-MF of 100 µT did not affect the decrease of proliferation rate or viability of menadione-treated human SH-SY5Y neuroblastoma cells.

Table 1 summarizes all relevant experimental conditions and outcomes of literature cited for ELF-MF-related effects on cancer cell response to differentiating or cytostatic/cytotoxic treatments.

**Table 1:** Main biophysical parameters of studies on extremely low-frequency magnetic fields (ELF-MF)-induced changes on cancer cell response to differentiating, cytostatic, or cytotoxic treatments.

Reference	ELF-MF treatment	Signal	B field direction	Device	MF-induced ΔT	Control	Cell model	Critical endpoint	Agent co-used	Interaction	Cancer-related behavior
Liburdy et al. (67)	12–50 Hz, 6.5 mT + DC	S	H	4-coil exposure system	NR	True sham (anti-parallel configuration)	Human MCF-7 breast cancer cells	Block of cell growth	Melatonin	Antagonism	☹
Harland and Liburdy (68)	60 Hz, 1.2 µT for 6 days	NR	H	4-coil exposure system	NR	NR	Human MCF-7 breast cancer cells	Block of cell growth	Tamoxifen (10^−7^ M)	Antagonism	☹
Chen et al. (51)	60 Hz, 4 µT	NR	V	4-coil exposure system	NR	True sham (anti-parallel configuration)	Friend erythroleukemia cells	Stimulation of differentiation	Dimethyl sulfoxide	Antagonism	☹
Chen et al. (51)	60 Hz, 4 µT	NR	V	4-coil exposure system	NR	True sham (anti-parallel configuration)	Friend erythroleukemia cells	Stimulation of differentiation	Hexamethylene bis-acetamide	Antagonism	☹
Laqué-Rupérez et al. (80)	25 Hz, 1.5 mT PEMF for 3 days	R	V	Coils in Helmholtz configuration	NR	Unenergized system	Human MCF-7 breast cancer cells	Block of cell growth	Methotrexate	None	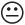
Ruiz-Gómez et al. (81)	1–25 Hz, 1.5 mT PEMF for 1 h	R	V	Coils in Helmholtz configuration	NR	Unenergized system	Human colon adenocarcinoma HCA cells	Block of cell growth	Mitomycin C	Potentiation	☺
Ruiz-Gómez et al. (81)	1–25 Hz, 1.5 mT PEMF for 1 h	R	V	Coils in Helmholtz configuration	NR	Unenergized system	Human colon adenocarcinoma HCA cells	Block of cell growth	Vincristine	Potentiation	☺
Ruiz-Gómez et al. (81)	1–25 Hz, 1.5 mT PEMF for 1 h	R	V	Coils in Helmholtz configuration	NR	Unenergized system	Human colon adenocarcinoma HCA cells	Block of cell growth	Cisplatin	Potentiation	☺
Pirozzoli et al. (69)	50 Hz, 1 mT for 1–3 days	NR	H	Coils in Helmholtz configuration	Actively avoided but not reported	True sham (anti-parallel configuration)	Human neuroblastoma LAN-5 cells	Stimulation of differentiation	Retinoic acid	Antagonism	☹
Pirozzoli et al. (69)	50 Hz, 1 mT for 1–3 days	NR	H	Coils in Helmholtz configuration	Actively avoided but not reported	True sham (anti-parallel configuration)	Human neuroblastoma LAN-5 cells	Stimulation of apoptosis	Camptothecin	Antagonism	☹
Ding et al. (59)	60 Hz, 5 mT for 24 h	NR	NR	Coils in Helmholtz configuration	NR	NR	Human leukemia HL-60 cells	Stimulation of apoptosis	Hydrogen peroxide	Potentiation	☺
Girgert et al. (70)	50 Hz, 1.2 mT for 7 days	S	NR	Coils	NR	NR	Human MCF-7 breast cancer cells	Stimulation of cell growth	Tamoxifen (<10^–6^ M)	Potentiation	☹
De Nicola et al. (82)	100 mT for 4 h	NR	NR	NR	Actively avoided but not reported	NR	Human U937 macrophage cells from histiocytic lymphoma	Stimulation of apoptosis	Puromycin	Antagonism	☹
Palumbo et al. (71)	Intermittent 50 Hz, 1 mT for 1 h	S	V	4-coil exposure system	NR	True sham (anti-parallel configuration)	Human Jurkat leukemic cells	Stimulation of apoptosis	Anti-Fas treatment	Antagonism	☹
Koyama et al. (83)	60 Hz, 5 mT for 2–24 h	NR	NR	Coils in Helmholtz configuration	NR	Unenergized system	Human glioblastoma A172 cells	Cell survival	Methyl methanesulfonate	None	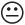
Koyama et al. (83)	60 Hz, 5 mT for 2–24 h	NR	NR	Coils in Helmholtz configuration	NR	Unenergized system	Human glioblastoma A172 cells	Cell survival	Hydrogen peroxide	None	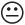
Jian et al. (60)	Intermittent 100 Hz, 0.7 mT for 1–3 h	S	V	Coils	NR	Unenergized system	Human BEL-7402 liver cancer cell line	Stimulation of apoptosis	X-ray irradiation	Potentiation	☺
Garip and Akan (84)	50 Hz, 1 mT for 3 h	NR	NR	Coils	Actively avoided but not reported	NR	Human erythroleukemia K562 cells	Stimulation of apoptosis	Hydrogen peroxide	Potentiation	☺
Marcantonio et al. (54)	50 Hz, 1 mT for 24–72 h	S	NR	Coils in Helmholtz configuration	NR	True sham (anti-parallel configuration)	Human neuroblastoma BE(2)C cells	Stimulation of differentiation	All-trans-retinoic acid	Potentiation	☺
Kaszuba-Zwoinska et al. (62)	Intermittent 50 Hz, 45 ± 5 mT PEMF for 9 h	NR	NR	NR	NR	Unrelated system	Human U937 macrophage cells from histiocytic lymphoma	Stimulation of apoptosis	Puromycin	Antagonism	☹
Kaszuba-Zwoinska et al. (63)	Intermittent 50 Hz, 45 ± 5 mT PEMF for 12 h	NR	NR	NR	NR	Unrelated system	Human acute monocytic leukemia MonoMac6 cells	Stimulation of apoptosis	Puromycin	Potentiation	☺
Kaszuba-Zwoinska et al. (63)	Intermittent 50 Hz, 45 ± 5 mT PEMF for 12 h	NR	NR	NR	NR	Unrelated system	Human acute monocytic leukemia MonoMac6 cells	Stimulation of apoptosis	Cyclophosphamide	Potentiation	☺
Kaszuba-Zwoinska et al. (63)	Intermittent 50 Hz, 45 ± 5 mT PEMF for 12 h	NR	NR	NR	NR	Unrelated system	Human acute monocytic leukemia MonoMac6 cells	Stimulation of apoptosis	Colchicine	Potentiation	☺
Trillo et al. (55)	50 Hz, 100 µT for 42 h	S	V	Coils in Helmholtz configuration	NR	Unenergized system	Human hepatocarcinoma HepG2 cells	Stimulation of cell growth	All-trans-retinol (0.5 × 10^−6^ M)	Antagonism	☺
Trillo et al. (55)	50 Hz, 100 µT for 42 h	S	V	Coils in Helmholtz configuration	NR	Unenergized system	Human neuroblastoma NB69 cells	Stimulation of cell growth	All-trans-retinol (0.5 × 10^−6^ M)	Potentiation	☹
Trillo et al. (56)	50 Hz, 10–100 µT for 42 h	S	V	Coils in Helmholtz configuration	NR	Unenergized system	Human neuroblastoma NB69 cells	Inhibition of cell growth	All-trans-retinol (2 × 10^−6^ M)	None	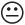
Cid et al. (72)	50 Hz, 10 µT for 90 h	S	V	Coils in Helmholtz configuration	NR	Unenergized system	Human hepatocarcinoma HepG2 cells	Block of cell growth	Melatonin	Antagonism	☹
Mansourian et al. (73)	93.25–159.4 µT for 10 min	NR	H	Coils in Helmholtz configuration	NR	NR	Human erythroleukemia K562 cells	Stimulation of apoptosis	Bleomycin	Antagonism	☹
Giorgi et al. (85)	50 Hz, 1 mT PEMF for 1–72 h	R	V	Coils in Helmholtz configuration	NR	True sham (anti-parallel configuration)	Human neuroblastoma BE(2)C cells	Stimulation of apoptosis	Hydrogen peroxide	None	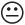
Mizuno et al. (78)	60 Hz, 5 mT for 24 h	S	V	Coils in Helmholtz configuration	NR	NR	Human SV40-transformed fibroblast	Cell survival	UV-B irradiation	None	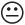
Mizuno et al. (78)	60 Hz, 5 mT for 24 h	S	V	Coils in Helmholtz configuration	NR	NR	Human SV40-transformed xeroderma pigmentosum cells	Cell survival	UV-B irradiation	None	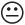
Jung et al. (57)	50 Hz, 1 mT for 5 days	S	V	Coils in Helmholtz configuration	NR	Unrelated system	Rat pheochromocytoma PC12 cells	Stimulation of differentiation	Nerve growth factor	Potentiation	☺
Wócik-Piotrowicz et al. (61)	35 Hz, 6.5 mT	S	V	Coils in Helmholtz configuration	±0.1°C, with no details	NR	Human U937 macrophage cells from histiocytic lymphoma	Stimulation of cell death	Puromycin	Potentiation	☺
Brisdelli et al. (74)	50 Hz, 1 mT for 24–72 h	S	V	Coils in Helmholtz configuration	±0.3°C	True sham (anti-parallel configuration)	Human erythroleukemia K562 cells	Block of cell growth	Quercetin	Antagonism	☹
Osera et al. (86)	75 Hz, 2 mT PEMF for 40 min	NR	V	Coils	NR	Unrelated system	Human neuroblastoma SH-SY5Y cells	Stimulation of cell death	Hydrogen peroxide	Antagonism	☹
Höytö et al. (79)	50 Hz, 100 µT for 24 h	S	H	Coils in Helmholtz configuration	NR	Unrelated system	Human neuroblastoma SH-SY5Y cells	Cell survival	Menadione	None	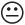
Kesari et al. (87)	50 Hz, 10–30 mT for 24 h	NR	H	Coils in Helmholtz configuration	NR	Unrelated system	Human neuroblastoma SH-SY5Y cells	Cell survival	Menadione	None	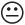
Kesari et al. (87)	50 Hz, 10–30 mT for 24 h	NR	H	Coils in Helmholtz configuration	NR	Unrelated system	Rat glioma C6 cells	Cell survival	Menadione	None	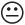
Falone et al. (76)	50 Hz, 1 mT for 15 days	S	H	Coils	<0.05°C, with no details	Unenergized system	Human neuroblastoma SH-SY5Y cells	Block of cell growth	Methylglyoxal	Antagonism	☹
Falone et al. (88)	75 Hz, 2 mT PEMF for 45 min	S	H	Coils	<0.05°C, with no details	Unenergized system	Human neuroblastoma SK-N-BE(2) cells	Stimulation of cell death	Hydrogen peroxide	Antagonism	☹
Baharara et al. (64)	50 Hz, 20 mT for 2 h	NR	NR	NR	NR	NR	Human ovarian adenocarcinoma A2780 cells	Stimulation of apoptosis	Cisplatin	Potentiation	☺
Benassi et al. (66)	50 Hz, 1 mT for 24 h	NR	H	Coils in Helmholtz configuration	±0.2°C	True sham (anti-parallel configuration)	Differentiated human neuroblastoma SH-SY5Y cells	Block of cell growth	1-methyl-4-phenylpyridinium	Potentiation	☺
Sanie-Jahromi et al. (89)	Intermittent 50 Hz, 0.50 mT for 30 min	S	H	Coils	NR	Unenergized system	Human MCF-7 breast cancer cells	Stimulation of cell death	Cisplatin + bleomycin	Potentiation	☺
Sanie-Jahromi et al. (89)	Intermittent 50 Hz, 0.50 mT for 30 min	S	H	Coils	NR	Unenergized system	Human neuroblastoma SH-SY5Y cells	Stimulation of cell death	Cisplatin + bleomycin	Potentiation	☺
Falone et al. (77)	50 Hz, 1 mT for 5–10 days	S	H	Coils	<0.05°C, with no details	Unenergized system	Human neuroblastoma SH-SY5Y cells	Block of cell growth	Doxorubicin	Antagonism	☹
Falone et al. (77)	50 Hz, 1 mT for 5–10 days	S	H	Coils	<0.05°C, with no details	Unenergized system	Human neuroblastoma SH-SY5Y cells	Block of cell growth	Hydrogen peroxide	Antagonism	☹
Akbarnejad et al. (90)	Square-wave 100 Hz, 10 mT for 6 days	S	V	Coils in Helmholtz configuration	Actively avoided but not reported	NR	Human glioblastoma U87 cells	Stimulation of apoptosis	Temozolomide	Potentiation	☺
Akbarnejad et al. (90)	Square-wave 100 Hz, 10 mT for 6 days	S	V	Coils in Helmholtz configuration	Actively avoided but not reported	NR	Human glioblastoma T98G cells	Stimulation of apoptosis	Temozolomide	Potentiation	☺

*H, horizontal; V, vertical; S, sinusoidal; R, rectangular; NR, not reported*.

*☹, ELF-MF-induced protection against differentiating, cytostatic, or cytotoxic treatments; ☺, ELF-MF-induced sensitization against differentiating, cytostatic, or cytotoxic treatments; 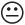, ELF-MF did not change cellular response to differentiating, cytostatic, or cytotoxic treatments*.

## ELF-MF-Related Effects on Cancer Cell Response to DNA-Damaging Challenges

DNA integrity is known to be a critical factor for correct cellular function and proliferation, therefore DNA-damaging agents have a long history in cancer chemotherapy (91). Not unexpectedly, the overexpression of DNA repairing capacity has been linked to the development of cancer chemoresistance (9). Over the past 15 years, several researchers have attempted to establish whether and how ELF-MF may have an impact on cancer cell response to DNA-targeting treatments.

The co-exposure of Jurkat leukemic cells to power frequency MF (1 mT) and 1,2,4-benzenetriol (1,2,4-BT) for 1 h enhanced significantly the 1,2,4-BT-induced DNA damage (92). Interestingly, the same authors showed also that the co-treatment of Jurkat cells with hydroquinone and the ELF-MF led to the appearance of a strong genotoxic effect that was not observed with the two treatments alone (92). By co-treating human UVW glioma cells with both ELF-MF (50 Hz, 1 mT for 12 h) and γ-radiation, Mairs et al. (93) revealed that ELF-MF can inhibit the repair of DNA lesions.

Conversely, many papers reported substantially different outcomes. Harris et al. (94) showed that γ-irradiation-induced blockade of G2 cell cycle in human adenocarcinoma HeLa cells was partially reverted by a 72-h exposure to a 50 Hz, 2 mT MF. In a recent study, Luukkonen et al. (95) evaluated the effects of a 24-h 50 Hz, 100 µT ELF-MF in combination with menadione, a free radical, and DNA damage inducer (96), on the level of DNA damage. In their work, Luukkonen et al. (95) established that the MF reverted to some extent the degree of DNA damage following the incubation with menadione. Sanie-Jahromi et al. (97) recently reported that in human neuroblastoma cells the repression of non-homologous end joining (NHEJ)-related genes associated with treatment with β-lapachone, a natural naphthoquinone anticancer drug (98), was totally antagonized by a subsequent exposure to an intermittent 50 Hz, 0.50 mT MF (30 min overall).

On the other hand, the co-exposure to ELF-MF and a carcinogenic compound may not have significant effects. Pasquini et al. (99) found that a 24-h 50 Hz, 5 mT MF did not alter the genotoxic damage in Jurkat cells incubated with known carcinogens like benzene, 1,4-benzenediol, and 1,2,4-benzenetriol.

Table 2 summarizes all relevant experimental conditions and outcomes of literature cited for ELF-MF-related effects on cancer cell response to DNA-damaging treatments.

**Table 2:** Main biophysical parameters of studies on extremely low-frequency magnetic fields (ELF-MF)-induced changes on cancer cell response to DNA damage-promoting treatments.

Reference	ELF-MF treatment	Signal	B field direction	Device	MF-induced ΔT	Control	Cell model	Critical endpoint	Agent co-used	Interaction	Cancer-related behavior
Harris et al. (94)	50 Hz, 2 mT for 72 h	NR	V	Coils in Helmholtz configuration	±0.1°C	Unenergized system	Human adenocarcinoma HeLa cells	G2 cell cycle blockade	γ-irradiation	Antagonism	☹
Pasquini et al. (99)	50 Hz, 5 mT for 24 h	S	NR	Coils	±0.3°C, with no details	Unenergized system	Human Jurkat leukemic cells	Genotoxic effect	Benzene	None	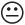
Pasquini et al. (99)	50 Hz, 5 mT for 24 h	S	NR	Coils	±0.3°C, with no details	Unenergized system	Human Jurkat leukemic cells	Genotoxic effect	1,4-benzenediol	None	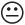
Pasquini et al. (99)	50 Hz, 5 mT for 24 h	S	NR	Coils	±0.3°C, with no details	Unenergized system	Human Jurkat leukemic cells	Genotoxic effect	1,2,4-benzenetriol	None	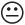
Moretti et al. (92)	50 Hz, 1 mT for 1 h	S	H	Coils in Helmholtz configuration	±0.3°C	True sham (anti-parallel configuration)	Human Jurkat leukemic cells	Genotoxic effect	1,2,4-benzenetriol	Potentiation	☺
Mairs et al. (93)	50 Hz, 1 mT for 12 h	S	H	Coils	Actively avoided but not reported	Unenergized system	Human glioma UVW cells	Genotoxic effect	γ-irradiation	Potentiation	☺
Falone et al. (32)	50 Hz, 1 mT for 4 days	S	H	Coils	<0.05°C, with no details	Unenergized system	Human neuroblastoma SH-SY5Y cells	Genotoxic effect	Hydrogen peroxide	Potentiation	☺
Koyama et al. (83)	60 Hz, 5 mT for 2–24 h	NR	NR	Coils in Helmholtz configuration	NR	Unrelated system	Human glioblastoma A172 cells	Genotoxic effect	Methyl methanesulfonate	Potentiation	☺
Koyama et al. (83)	60 Hz, 5 mT for 2–24 h	NR	NR	Coils in Helmholtz configuration	NR	Unrelated system	Human glioblastoma A172 cells	Genotoxic effect	Hydrogen peroxide	Potentiation	☺
Bułdak et al. (7)	50 Hz, 1 mT for 16 min	NR	H	Coils	±0.4°C	Unrelated system	Murine squamous cell carcinoma AT478 cells	Genotoxic effect	Cisplatin	Antagonism	☹
Bułdak et al. (7)	50 Hz, 1 mT for 16 min	NR	H	Coils	±0.4°C	Unrelated system	Murine squamous cell carcinoma AT478 cells	Genotoxic effect	Hydrogen peroxide	Antagonism	☹
Luukkonen et al. (100)	50 Hz, 100 µT for 24 h	NR	H	Coils in Helmholtz configuration	NR	Unrelated system	Human neuroblastoma SH-SY5Y cells	Genotoxic effect	Menadione	None	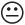
Giorgi et al. (85)	50 Hz, 1 mT PEMF for 1–72 h	R	V	Coils in Helmholtz configuration	NR	True sham (anti-parallel configuration)	Human neuroblastoma BE(2)C cells	Genotoxic effect	Hydrogen peroxide	None	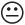
Kesari et al. (101)	50 Hz, 100 µT for 24 h	NR	H	Coils in Helmholtz configuration	NR	Unrelated system	Human neuroblastoma SH-SY5Y cells	Genotoxic effect	Menadione	None	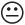
Kesari et al. (87)	50 Hz, 30 µT for 24 h	NR	H	Coils in Helmholtz configuration	NR	Unrelated system	Human neuroblastoma SH-SY5Y cells	Genotoxic effect	Menadione	Potentiation	☺
Kesari et al. (87)	50 Hz, 30 µT for 24 h	NR	H	Coils in Helmholtz configuration	NR	Unrelated system	Rat C6 glioma cells	Genotoxic effect	Menadione	None	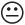
Sanie-Jahromi et al. (89)	Intermittent 50 Hz, 0.50 mT for 30 min	S	H	Coils	NR	Unenergized system	Human MCF-7 breast cancer cells	Down-regulation of DNA repair	Cisplatin	Potentiation	☺
Sanie-Jahromi et al. (89)	Intermittent 50 Hz, 0.50 mT for 30 min	S	H	Coils	NR	Unenergized system	Human neuroblastoma SH-SY5Y cells	Down-regulation of DNA repair	Cisplatin	Potentiation	☺
Luukkonen et al. (95)	50 Hz, 100 µT for 24 h	NR	H	Coils in Helmholtz configuration	NR	Unrelated s ystem	Human neuroblastoma SH-SY5Y cells	Genotoxic effect	Menadione	Antagonism	☹
Sanie-Jahromi et al. (97)	Intermittent 50 Hz, 0.50 mT for 30 min	S	H	Coils	NR	Unenergized system	Human neuroblastoma SH-SY5Y cells	Down-regulation of DNA repair	β-Lapachone	Antagonism	☹

*H, horizontal; V, vertical; S, sinusoidal; R, rectangular; NR, not reported*.

*☹, ELF-MF-induced protection against DNA-damaging treatments; ☺, ELF-MF-induced sensitization against DNA-damaging treatments; 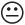, ELF-MF did not change cellular response to DNA-damaging treatments*.

## ELF-MF-Related Effects on Cancer Cell Antioxidative/Detoxification Responses

Cancer cell death-inducing chemical or physical treatments is often based on genotoxic damage through severe redox imbalance and overproduction of ROS (102). In general, cancer cells exhibit over-protection against ROS through high expression of critical antioxidant enzymes, and this seems to promote tumor progression and MDR (103–107). For example, both high-glutathione peroxidase (GPX) and catalase activity, along with increased level of reduced glutathione (GSH) are linked to MDR phenotype in some cancer cells (108–112). Accordingly, many chemotherapies act to overwhelm the high-ROS-scavenging capacity of tumors and cancers (102, 107, 113, 114). Unfortunately, most studies were carried out by monitoring ELF-MF-induced effects on redox-related pathways, without elucidating whether EMF-MF could potentiate or depress oxidative stress induced by pro-oxidant stimuli in tumor cell lines (115).

In human SH-SY5Y neuroblastoma cells, a 24 h a 50 Hz, 1 mT ELF-MF exposure significantly limited the H_2_O_2_-induced increase of catalase, whose catalysis ensures a rapid removal of hydrogen peroxide from the cell (116). In addition, cytochrome P450 (CYP450) and the glutathione S-transferase (GST), which are two crucial detoxification enzymes that have been known for a long time to be responsible for resistance to anti-tumor drugs (117), were reported to be activated by a 50 Hz, 1 mT ELF-EMF (32, 116). As shown by Patruno et al. (118), the effect of phorbol 12-myristate 13-acetate (PMA) on the activity of CYP450 was enhanced after human erythro-leukemic cells were exposed to an acute sinusoidal 50 Hz, 1 mT MF. We have recently demonstrated that human neuroblastoma cells respond to a long-term 50 Hz, 1 mT ELF-MF by increasing CAT- and GPX-based scavenging activities, as well as by improving the availability of reduced glutathione (76, 77). Remarkably, GSH is known to act as an essential co-factor for both antioxidant GPX and phase II drug-metabolizing GST enzymes (119–121). In addition, GSH is the essential co-factor for the entry of MG into the glyoxalase-mediated cycle, a detoxification pathway highly overexpressed in many malignancies (25). Accordingly, we recently found that a long-term ELF-MF exposure increases the efficiency of MG-targeting enzymatic systems, with the aim of suppressing the accumulation of the cancer-static glycolytic metabolite in hyperproliferating cancer cells, and this in turn was linked to a strongly reduced vulnerability to treatments with exogenous MG (76). We also showed that ELF-MF-exposed neuroblastoma cells exhibit activation of sirtuin 3 expression (77), and SIRT3-dependent signaling has been recently linked to cancer metastatic development, through the improvement of mitochondrial integrity and fitness in response to oxidative proteotoxic stress (122). Moreover, some of us (77) have also demonstrated that a 50 Hz, 1 mT ELF-MF exposure upregulated the nuclear translocation of the erythroid 2-related nuclear transcription factor 2, which has been recently linked to the acquisition of resistance against ROS-promoting anticancer drugs in several cancer cell types (105, 106, 123, 124).

However, literature reports also findings that suggest that ELF-MF are able to diminish the antioxidant/detoxificant-based response of cancer cells to redox-based challenges. Patruno et al. (125) found that a 50 Hz, 1 mT MF decreased the degree of lipopolysaccharide (LPS)-induced activation of the master regulator of phase I metabolism CYP450 in human monocytic leukemia cells. De Nicola et al. (82) described how lymphoblasts from human histiocytic lymphoma underwent a strong decrease of reduced glutathione levels upon 2-h treatment with 50 Hz MF, in parallel with a marked elevation of ROS concentration. On the other hand, Benassi et al. (66) demonstrated that cytotoxic effects elicited by MPP^+^ (1-methyl-4-phenylpyridinium) were significantly potentiated when SH-SY5Y cells were pre-exposed to a 24-h ELF-MF, and this was linked to the ELF-MF-related induction of a massive oxidative stress. In support of this view, the addition of permeable thiol-based antioxidants like *N*-acetyl-l-cysteine or GSHest was able to limit the MF-induced increase in MPP^+^-dependent apoptotic response (66).

It should be highlighted that papers claiming that ELF-MF do not elicit significant effect on cancer cell response to redox-active treatments are not rare. Fiorani et al. (126) did not find any MF-dependent alteration of redox environment in human erythroleukemia K562 cells after treatment with DNA-damaging agent methyl methanesulfonate (MMS). Patruno et al. (125) found that a 50 Hz, 1 mT ELF field did not affect the response of catalase enzymatic function to the treatment with bacterial LPS, a major inducer of systemic inflammatory reactions and oxidative stress (127). Patruno et al. (118) also demonstrated that the effect of tumor promoter PMA on catalase enzymatic defense of human erythro-leukemic cells was not affected by an acute sinusoidal 50 Hz, 1 mT MF.

## Considerations on Multiple Endpoint-Based Studies

The deep understanding of how non-invasive external stimuli such as ELF-MF may modulate cancer cell phenotype and response to chemical/physical treatments represents an ever-increasing research topic with hundreds of scientific teams all around the globe committed to investigate the details of molecular interaction between ELF fields and cancer cells. Such studies might contribute to determine whether cancer cell phenotype may be ameliorated or worsened by the exposure to physical factors that are becoming increasingly common in our electricity-based modern life.

Surprisingly, only one work was carried out by considering several levels of the complex phenomenon. Specifically, Kesari et al. (87) investigated how human neuroblastoma SH-SY5Y cells and rat glioma C6 cells responded to menadione after 24-h exposure to power frequency, low-intensity MF in terms of cell viability, DNA damage, and intracellular ROS. The authors observed that the modulation by the MF of menadione-induced responses were different in the two cell lines, with no clear correlation among the degree of cell death, genotoxic damage, and the mitochondrial superoxide production. Much more studies considered two out of three major endpoints on which this review is focused. Some of us (32) reported that a 96-h power frequency, 1 mT MF enhanced the DNA-damaging power of hydrogen peroxide on human neuroblastoma cells, and this was due to the MF-dependent increase of H_2_O_2_-related ROS overproduction. Similarly, Garip and Akan (84) showed that a 3-h 50 Hz, 1 mT MF potentiated the H_2_O_2_-induced apoptotic response of erythroleukemia cells, and this was directly correlated to the MF-dependent elevation of HSP70 and ROS following the pro-oxidant insult. This finding gave a strong impulse to research as many tumor cells are known to express constitutively elevated amounts of heat-shock proteins, as a means through which chemotherapeutic resistance and increased tumorigenesis may be developed (128, 129). Sanie-Jahromi et al. (89) found that a 30-min exposure to an intermittent 50 Hz, 0.50 mT MF potentiated the cell death-promoting action of the combined treatment with cisplatin + bleomycin on human breast cancer cells and neuroblastoma cells, and this was likely to be due to the MF-related potentiation of the repressing effect elicited by anticancer treatment on genes related to NHEJ, a critical pathway for repairing DNA double-strand breaks (130). On the other hand, some of us showed that a 40-min pulsed 75 Hz, 2 mT MF protects two lines of human neuroblastoma cells from hydrogen peroxide-dependent cell death, and this was found to be related to a significant overprotection from mitochondrial ROS production (86, 88), thus linking the loss of cytotoxic effect of H_2_O_2_ to the MF-dependent activation of the major antioxidant defense systems within the mitochondrion. These data are supported by findings of others that found elevated MnSOD mRNA expression following intermittent exposure to 50 Hz, 0.50 mT ELF-MF for 30 min (131). Such experimental observations are gaining remarkable importance since in many tumor types SOD2 levels were found to increase as early-stage non-invasive disease advances to late-stage malignancy (132). As the energy supply from the glycolytic pathway may not fulfill the increased energy requirements of aggressive growth, particularly in the later stages of carcinogenesis, highly malignant cancer cells could need to increase the efficiency of mitochondrial function and protect the mitochondrial environment from ROS-based macromolecular damage (133). Such hypothesis is supported by our data that showed an increased mitochondrial activity following a long-term exposure to power frequency ELF-MF (76). In neuroblastoma cells, Kesari et al. (101) found that a 24-h 50 Hz, 100 µT MF antagonizes menadione-dependent increase of lipid peroxidation, although this did not affect micronuclei frequency, thus suggesting that the MF-related redox modulation may not have significant biological relevance, at least in terms of accumulation of genotoxic effects. Bułdak et al. (7) found that cisplatin-dependent genotoxicity and oxidative damage in murine carcinoma cells were reverted by an acute exposure to 50 Hz, 1 mT MF, and this was paralleled by an MF-induced improvement of MnSOD superoxide-scavenging activity, thus providing evidence of a remarkable protective effect of ELF fields on the mitochondrial oxidative load derived from the use of a clinically relevant antiblastic drug on cancer cells. Such findings were not confirmed by studies on neuroblastoma cells that seem not to be particularly affected by a mid-term treatment with a low-intensity ELF-MF as regard to menadione-induced redox imbalance. In particular, Luukkonen et al. (100) found that the menadione-induced oxidative stress was increased in neuroblastoma cells in after exposure to a 24-h 50 Hz, 100 µT ELF-MF, even though the genotoxic effect of menadione was not significantly affected by the MF exposure, thus suggesting that the pro-oxidant effect of MF was not sufficient to induce any additional hazard to menadione-dependent genomic injury. Moreover, Giorgi et al. (85) reported that a pulsed 50 Hz, 1 mT MF did not protect human neuroblastoma BE(2)C cells against the apoptotic effect of hydrogen peroxide, and this was confirmed by the lack of any significant effect of the PEMF on the H_2_O_2_-dependent increase of γ-H2AX-positive nuclei, thus suggesting that pulsed ELF fields may elicit different effects on neuroblastoma cells depending on physical parameters of the applied MF.

In human glioblastoma cells, Koyama et al. (83) did not find any link between the MF-induced potentiation of the genotoxic effect of pro-oxidants and the cytotoxic effect of both MMS and hydrogen peroxide. Taken together, such findings seem to suggest that MF can elevate the effects of DNA-damaging redox-active agents without affecting significantly cell viability or growth, and this could induce genomic instability and the accumulation of further genetic mutations, thus allowing cancer cells to accumulate further oncogenic mutations in order to progress toward increased aggressiveness (134). Interestingly, Akbarnejad et al. (90) demonstrated that the cytotoxic effect of temozolomide on two lines of human glioblastoma cells was enhanced by a 6-day square-wave 100 Hz, 10 mT MF, and this was associated with the potentiation of the TMZ-induced activation of oxidative stress-responsive genes. However, the authors failed to demonstrate that such a response was dependent on a different modulation of TMZ-induced ROS overproduction.

## Concluding Remarks

As evident from the above discussed results, many contradictory effects of ELF-MF were reported in literature. This inevitably raises the question as to what could lead to such discrepancies. Conflicting data might be due to differences in type of cell lines or cell lineage used. Such variability was already discussed by Myrtill Simkó’s group in 2007. It must be also considered that important variables related to the exposure conditions vary greatly from study to study (i.e., flux density and duration), even though the most common wave forms and frequencies undoubtedly reflect the characteristics of power frequency-generated ELF-MFs worldwide (i.e., sinusoidal and 50/60 Hz). In addition, different laboratory methods and techniques may also lead to contradictory results. Furthermore, it should not be ignored that sources of artifacts have been recognized to involve real-sham inclusion, mechanical vibrations of the sample holder, and small temperature differences in the media (135). With regard to thermal effects, despite the fact that cancer cells are more vulnerable to high temperatures than normal cells (136, 137), recent findings seem to indicate that an increase in temperature could enhance cancer cell resistance to known antiblastic compounds, such as cisplatin (138, 139). In this review, we show that the great majority (61%) of results were reported by studies in which no clear check of MF-induced heating effect was reported. Moreover, less than 12% of researches were conducted by avoiding actively (e.g., liquid cooling) any MF-dependent temperature change in the samples.

Surprisingly, less than 20% of the experimental designs considered true-sham conditions as the adequate reference controls. 47% of the papers either did not report how the controls were handled or did use an unrelated system to host not-exposed cells (for example, a similar incubator with no exposure system). The relative majority of investigations (34%) were conducted considering cells hosted in unenergized systems (e.g., disconnected coils) as the “sham” condition.

All the considerations above raise the need for much stronger efforts to set uniform standards for the experimental community, and to minimize sources of lab-to-lab variability, in order not to introduce systematic bias that would critically alter the cellular responses.

As mentioned above, Our review of the existing literature on the field show that most studies used sinusoidal 50/60 Hz ELF fields, with only few papers dealing with square-wave signal. With regard to MF direction, vertical polarization was highly prevalent in investigations aimed at discovering ELF-MF-induced effects on cancer cell response to cytotoxic/differentiating treatments, whereas horizontal B fields were mainly used in studies dealing with MF-related effects on cancer cell response to redox-active treatments and DNA-damaging agents.

Surprisingly, the percentage of studies with no clear indication of B field direction was relevant (14–26%, depending on the endpoint studied). The promotion of a more resistant phenotype against cytotoxic/differentiating treatment was mainly reported by authors that used a horizontal B field (64%), whereas when B direction was either vertical or not reported, results were more controversial.

Taking together all the results obtained by researcher groups over the past 25 years, *in vitro* co-exposure- or conditioning-based studies seem to indicate that ELF-MF modify significantly how malignant or tumor cells respond to pro-differentiating or cytotoxic/cytostatic treatments. Most of the studies were carried out using human breast cancer and neuroblastoma cells, even though leukemic, hepatocarcinoma, and glioblastoma were also used as malignant *in vitro* model. Most of the works available provided evidence that tumor/cancer cells exhibit increased tolerance to chemical or physical treatments aimed at promoting differentiation and/or apoptotic cell death, with only few papers claiming that ELF-MF do not elicit significant effect on cancer cell behavior in response to cytotoxic or pro-differentiating treatment. More specifically, the great majority (>56%) of researches provided convincing evidence that power frequency ELF-MF (i.e., 50–60 Hz) are able to protect breast cancer cells and neuroblastoma cells from chemical treatments aimed at either targeting cell survival or promoting cell differentiation, especially for ELF-MF exposure duration of several days. Even if more sporadic, studies seem to suggest that short-term ELF-MF exposures may not affect the response of glioblastoma/glioma cells to pro-apoptotic inducers, but longer treatments could potentiate the cytotoxic effect of cell death-promoting compounds. Rare literature reports seem to indicate that the resistance of liver cancer cells to treatments aimed at targeting cell survival do not allow to draw any conclusion or evident hypothesis. Similarly, studies on human leukemic cells are not consistent, with some papers (43%) reporting MF-induced enhancement of the efficacy of either pro-apoptotic or differentiating treatments, and others (53%) showing the opposite effect. Interestingly, it should be highlighted that no study on leukemic cells reported a null effect of MF on cytotoxic treatments. Investigations on ELF-MF-induced effects on adenocarcinoma cells are scarce; however, the existing studies reported that short ELF-MF exposure increased the cytotoxic effect of common anticancer drugs, such as cisplatin and vincristin. Data on human macrophage cells from histiocytic lymphoma suggest that PMC-induced apoptosis may be reverted by short-term exposure to ELF-MFs.

Findings concerning the capacity of ELF-MF to affect cancer cell response to pro-differentiation chemical treatments are rare, and no clear conclusion may be drawn from the controversial data found.

With regard to how ELF-MF may alter cancer cell response to DNA-damaging agents, a high heterogeneity in terms of MF intensity applied, exposure protocols, and cell types used exists, however, the available results suggest that specific exposure paradigms are able to alter the genotoxic capacity of some compounds in certain cell models. We discussed above how more resistant phenotypes against cytotoxic/differentiating treatments were mainly reported in studies with horizontal B fields. However, horizontal ELF-MF seem not to render cancer cells more resistant against genotoxic challenge. Indeed, only 27% of the investigations conducted with horizontal MF showed ELF-MF-induced increase of cell resistance to DNA-damaging agents. The relative majority of the available studies found that ELF-MFs increased the effects of genotoxic compounds or physical factors on tumor/cancer cells. One-third of the reviewed papers reported a null effect of MF exposures on cancer cell response to DNA-targeting treatments. On the other hand, all the reports regarding the genotoxicity of chemical/physical challenge in carcinoma cells showed that ELF-MF exposure exerted a cytoprotective action. Lastly, magnetic flux densities and exposure duration seem not to be obvious determinants of the genotoxic response of cancer cells to DNA-damaging chemicals or ionizing radiation.

All the available results suggest that different cancer cell types react differently to ROS-perturbing ELF-MF, maybe because of their tissue-specific lineage, and such variability was already foreseen or discussed by Myrtill Simkó’s group in 2007 and 2014. Some studies seem to show that ELF-MF may trigger in cancer cells the activation of pathways aimed at scavenging more effectively ROS overproduction, thus providing additional defense against forthcoming treatments promoting oxidative cellular damage. Conversely, other researches provided evidence that ELF-MF may lower the tolerance of cancer cells toward additional oxidative-based challenges. Hence, some reports seem to suggest that the combined exposure to ELF-MF and other oxidative stress-inducing chemical or physical treatments may result in an increased oxidative damage and functional impairment. More specifically, ELF-MF treatment do not seem to exert any evident effect on the antioxidant response of human leukemic cells undergoing treatments with chemical tumor promoters. Conversely, ELF-MF are likely to enhance mitochondrial antioxidant defense in human neuroblastoma cells, and this is especially true for pulsed low-intensity or long-term exposures. Less information is available with regard to glia-derived cancer cells, even though the only two papers available report that ELF-MF appeared to sensitize malignant cells toward redox-active antiblastic drugs.

In summary, despite the increasing scientific interest in this topic, it seems extremely difficult to draw any definitive conclusion from the review of the existing literature in this field. Given the limited amount of specific interventional experiments based on *in vitro* co-exposures designs, and due to the highly controversial results, too many aspects remain hidden behind the open debate on how ELF-MF can affect cancer cell behavior or resistance against relevant antiblastic treatments, especially when the questions come to molecular mechanisms involved. For this reason, urgent further research is needed in order to define accurately the biomolecular mechanisms involved in the interaction between ELF fields and biosystems. In particular, careful attention should be paid with regard to the exact pathways that may be activated or repressed by ELF-MFs in tumor and cancer cells, as such processes may reveal molecular targets that, if appropriately confirmed *in vivo*, could help to develop innovative approaches aimed at controlling the fate of malignant cells.

## Future Perspectives

Growing evidence suggests that therapy resistance, relapse, and poor prognosis in human malignancies may be also driven by cancer stem cells (CSCs), a sub-population of highly undifferentiated tumor-initiating cells with self-renewal features and extreme drug-resistance capacity (3, 9). Unfortunately, findings concerning how the electromagnetic fields may affect adult stem cell biology are inconsistent (140). In particular, no data from *in vitro* or *in vivo* studies are available with regard to ELF-MF-induced effects on stimulation or depression of CSCs, with such research topic being totally unexplored so far.

Cancer stem cells seem to present substantially different metabolic requirements, with respect to cancer cells with not stemness characteristics, along with a remarkable DNA repair activity (1). In particular, growing evidence shows that CSCs produce more ATP than their differentiated progeny, as a reflection of enhanced mitochondrial function and metabolic activity (141–143). In addition, molecular switches within the mitochondrion seem to act as a crucial mediator in determining tumor metastatic dissemination (144, 145). Moreover, CSCs are endowed with a more powerful antioxidant defense system compared with their progeny, and such antioxidative trait helps in maintenaning low-ROS levels, along with the stemness and tumorigenic capacities of CSCs, thus leading to chemoresistance (1, 146–148). Some of us have already shown that neuroblastoma cells respond to long-term exposure to an ELF-MF by: (a) shifting metabolic supply from glycolysis to mitochondrial respiration (76); (b) activating the major ROS-targeting antioxidant systems and protecting protein and DNA from oxidative damage (77); and (c) enhancing mitochondrial protection from ROS-dependent damage (88).

On this basis, it becomes clear that extensive *in vitro* and *in vivo* studies aimed at verifying whether exposures to ELF-MF are able to confer specific survival advantage to CSCs, thus affecting the fate and behavior of CSCs, are crucially needed. In this context, recent evidence seems to suggest that epigenetic aberration may be involved in the acquisition of CSC-like properties (149), and acetylation is emerging as one of the most crucial chromatin modification, which occurs *via* highly regulated enzymatic processes, in which histone deacetylases (HDACs) appear to play a critical role (150). Class III HDACs, also referred to as sirtuins (SIRT1-7), are homologous to the yeast Sir2 family proteins and require coenzyme NAD^+^ for catalysis (151). Interestingly, our group has recently reported that a 50 Hz, 1 mT MF exposure increased protein expression of both SIRT1 and SIRT3, thus suggesting the possible MF-dependent activation of chromatin remodeling by nuclear and mitochondrial sirtuins (77).

Increased efflux of drugs by membrane transporters is another important mechanism through which cancer cells are able to develop MDR (152). To date, only a study was conducted to verify whether the exposure to an ELF field may result in the alteration of the expression pattern of the major MDR-related transporter P-glycoprotein (also called MDR1) in cancer cells. In particular, Walter et al. (153) found that the 1-beta-d-arabinofuranosylcytosine-induced up-regulation of MDR1 in H9 human leukemia cells was inhibited by a 17-h exposure to 60-Hz sinusoidal EF. Such investigations may possibly reveal that a common physical component of the human modern life may cause startling effects on a critical homeostatic feature of cancer cells, from which major challenges to effective/successful chemotherapy in cancer treatment may arise.

## Author Contributions

SF, FA, SS, and VC retrieved literature reports and wrote the main sections of the manuscript. MC, GE, and CT helped to write the manuscript. All authors reviewed the manuscript.

## Conflict of Interest Statement

The authors declare that the research was conducted in the absence of any commercial or financial relationships that could be construed as a potential conflict of interest. The reviewer SF and handling editor declared their shared affiliation. The handling Editor also declared a shared affiliation, though no other collaboration, with one of the authors [FA].
